# Excessive proinflammatory cytokine and chemokine responses of human monocyte-derived macrophages to enterovirus 71 infection

**DOI:** 10.1186/1471-2334-12-224

**Published:** 2012-09-21

**Authors:** Xun Gong, Jianfang Zhou, Wenfei Zhu, Na Liu, Jinsong Li, Lili Li, Yu Jin, Zhaojun Duan

**Affiliations:** 1 School of Basic Medical Sciences, Lanzhou University, Lanzhou, 730000, PR China; 2 National Institute for Viral Disease Control and Prevention, China CDC, Beijing, 100052, PR China; 3 Nanjingn Children’s Hospital, Medical School of Nanjing University, Nanjing, 210093, PR China

**Keywords:** Enterovirus 71, Macrophages, Proinflammatory cytokines, Chemokines, Toll-like receptors

## Abstract

**Background:**

The levels of proinflammatory cytokine or chemokine in blood and cerebrospinal fluid are thought to be one of predictors for clinical severity of enterovirus 71 (EV71) infection, yet the cellular sources or signalling mechanism remain undefined. Here, we focused on the response of human primary monocyte-derived macrophages (MDMs) to EV71 virus and its possible mechanisms.

**Methods:**

Human primary MDMs were infected by EV71 virus *in vitro*. Infectivity and viral replication were assayed, and cytokine responses were determined by Cytometric Bead Array(CBA) analysis. The relative changes of Toll-like receptors, retinoic acid-inducible gene I (RIG-I) and melamoma differentiation associated gene 5 (MDA5) mRNA expression were detected by real-time RT-PCR.

**Results:**

Effective infection and viral replication were detected in EV71-infected MDMs. The titters of progeny virus released from EV71-infected MDMs gradually increased from 6-h to 48-h point of infection (POI.). Proinflammatory cytokines: IL-1, IL-6, TNF-α but not IFN-α and γ were induced in MDMs by EV71. EV71 infection significantly increased the release of IL-8, IP-10 and RANTES at 12-h or 24-h POI. Upregulation of TLR2, TLR7 and TLR8 mRNA expression rather than TLR3, TLR4, TLR6, TLR9, TLR10, RIG-I, MDA5 were found at different time points in EV71-infected MDMs.

**Conclusions:**

Our findings suggested that macrophages are not only the important target cells but also the effectors during EV71 infection, and they may play an important role in the pathogenesis of EV71 infection. And the proinflammatory cytokine and chemokine responses in EV71-infected MDMs may be mediated by the activation of differential pattern of TLRs.

## Background

Enterovirus 71, a positive-stranded RNA virus, is highly infectious and can cause hand-foot-and-mouth disease (HFMD), herpangina, neurological diseases with potentially more serious complications such as encephalitis, aseptic meningitis, brain stem encephalitis (BE) in infants and young children. Many of patients died from fulminant pulmonary edema (PE) or hemorrhage, which was based on nervous system injury [1,2]. In recent years, its prevalence in Malaysia, Taiwan, Singapore, China, Korea and so on, and continuous spreading widely provoked global concern [3-7].

Although the pathogenesis of neurogenic PE caused by EV71 remains unclear, host factors especially host immune response rather than EV71 itself or its genotype may be one of important determinants for the disease severity [1,8]. Excessive proinflammatory cytokine and chemokine responses were thought to contribute to the severity of EV71 infection [9]. Current findings implied that the inflammatory cytokines or chemokines are probably synthesized by infiltrated mononuclear cells (macrophages or T cells) in tissues or neuron-surrounding cells, such as microglia (the resident macrophages in central nervous system (CNS)) or astrocytes in EV71 infection [9-11]. Since monocyte-derived macrophages and microglia are derived from the common precursors in the bone marrow, express similar surface markers and perform roughly similar functions [12], both of them may be involved in the immune response to EV71 infection.

Previous studies have identified that the immune cells such as human peripheral blood mononuclear cells, human T cell line (Jurkat), monocytic cells, human immature dendritic cells can be infected with EV71 [13-15]. Macrophages play an important role in the innate immunity system, however, the interaction between macrophages and EV71 remains unknown. Furthermore, Toll-like receptors (TLRs) and RIG-I-like helicases (RLHs) recognize a number of viruses resulting in the activation of an innate immunity response that induce secretion of proinflammatory cytokines and chemokines [16-19]. In this study, we focused on the proinflammatory cytokine and chemokine responses of MDMs to EV71 infection. And we also investigated whether TLRs and RLHs were involved in EV71-infected MDMs, and explored the possible mechanisms of inflammatory responses to EV71 infection.

## Results

### Effective infection and viral replication of EV71 in human MDMs

**Figure 1:**
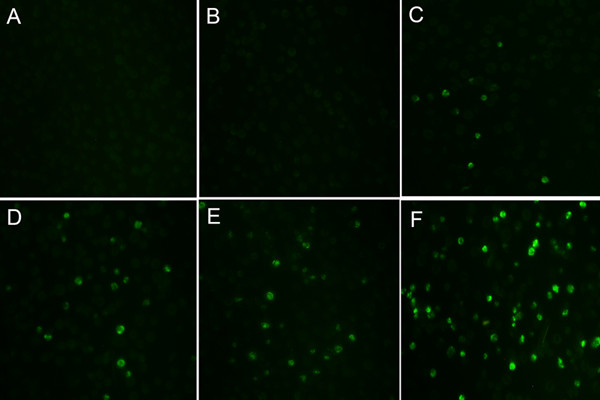
**A dose-dependent infection of EV71 virus in human monocyte-derived macrophages (MDMs).** Immunofluorescence staining of EV71 VP1 in mock- or EV71-infected MDMs was assayed at 24-h POI. **A**. mock, **B**. UV-inactivated EV71 infection, **C**. EV71 infection at a MOI of 0.1, **D**. EV71 infection at a MOI of 0.5, **E**. EV71 infection at a MOI of 1, **F**. EV71 infection at a MOI of 5.

**Figure 2:**
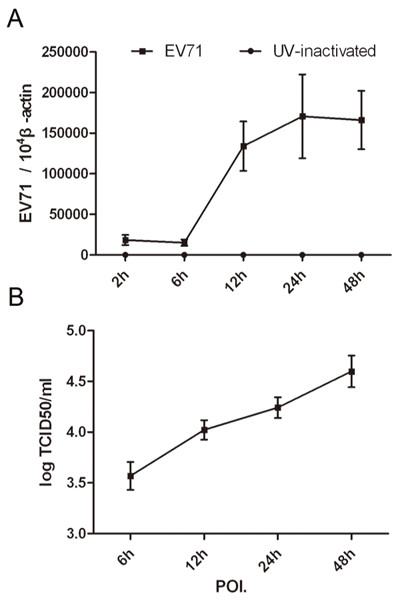
**Viral replication of EV71-infected human MDMs.** Viral gene copies were quantified by real-time RT-PCR. Supernatants from infected cells were collected at designated time points post inoculation of EV71 at a MOI of 5 and the viral titter was measured by TCID50 analysis on Vero cells. **A**. the change of VP1 gene. The number of gene copy was normalized to 10^4^copies of β-actin and expressed as mean ± standard error (SEM) from eight independent experiments, and each experiment was performed in triplicates. **B**. viral titters of EV71-infected MDMs cultures. The data were expressed as mean ± SEM from 8 independent experiments.

We first determined the infectivity of MDMs to EV71 by immunofluorescence staining of viral VP1. As evidenced by the expression of viral VP1^+^ at 12-h POI. (data not shown), human MDMs could be infected by EV71 virus. Furthermore, the infection showed a dose-dependent for infectious dose, as evidenced by an increase of VP1 expression at 24-h POI. along with a MOI of 0.1, 0.5, 1 and 5. (Figure 1C-F). The VP1+ fluorescence signal was detected in the cytoplasm in EV71-infected MDMs at 24-h POI., but not in mock or UV-inactivated EV71-infected MDMs at MOI of 5(Figure 1). Then the viral gene copy was quantified. An increase of viral gene copy was detected between 6-h and 12-h POI. (range from 16,421 ± 5061 to 136,027 ± 54,473 copies/10^4^ copies of beta-actin, *p = 0.008*, n = 8). The viral gene copies maintained at a high level at the following assessed time points, 24-h and 48-h POI, but showed no significant difference from that at 12-h POI. *(p = 0.194*, *p = 0.273*, n = 8) (Figure 2A). To identify whether MDMs produce infectious progeny particles or not, virus titers of culture supernatants were performed by measuring the 50% tissue culture infective dose (TCID50) on Vero cells and calculated by using the Reed and Muench formula [20]. The results showed that MDMs were productively infected by EV71 with approximate 10-fold increase in virus titter by 48-h POI. (Figure 2B, n  = 8). Our *in vitro* findings suggested that MDMs are susceptible to EV71 infection and maybe one of target cells *in vivo* during EV71 infection.

### Enhanced proinflammatory cytokine responses of human MDMs to EV71

**Figure 3:**
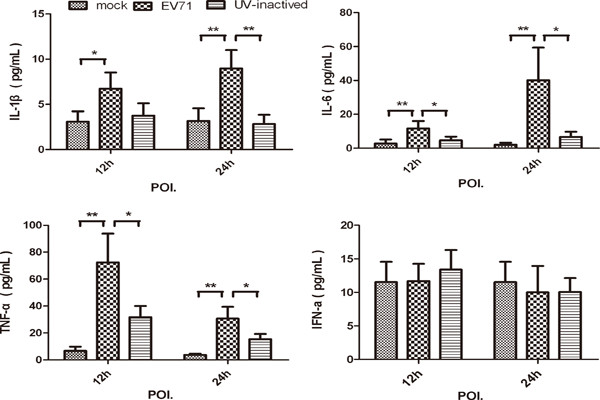
**Proinflammatory cytokines IL-1β, IL-6 and TNF-α induced in EV71 infected MDMs.** MDMs were infected with or without EV71 (live or UV-inactivated) at MOI of 5. Culture supernatants were harvested at 12-h and 24-h after infection to measure the indicated cytokines by CBA as described in Methods section. Data are expressed as mean ± SEM from eight independent experiments performed in triplicates. (*, *P < 0.05* and **, *P < 0.01*).

We subsequently determine the functional response of human MDMs to live EV71 or UV-irradiated EV71. Significant higher level of TNF-α was rapidly induced by live EV71 infection other than mock or UV-irradiated EV71 at the 12-h POI. (72.34 ± 52.56 pg/ml vs. 6.68 ± 7.67 pg/ml, 31.74 ± 20.26 pg/ml, respectively, *p = 0.001*, *p = 0.011*, n = 8), and then gradually decreased over time (30.68 ± 21.50 pg/ml, at 24-h POI.). Both IL-1β and IL-6 from MDMs were triggered by live EV71, and maintained a higher level at 12-h (*p = 0.018*, *p = 0.008*, n = 8) and 24-h POI. (*p = 0.007*, *p = 0.006*, n = 8) as compared with that of mock (Figure 3). In addition, we found that EV71 infection failed to induce IFN-α releasing from human MDMs, and the IFN-γ was undetectable.

### Enhanced production of IL-8, RANTES and IP-10 from human MDMs infected with EV71

**Figure 4:**
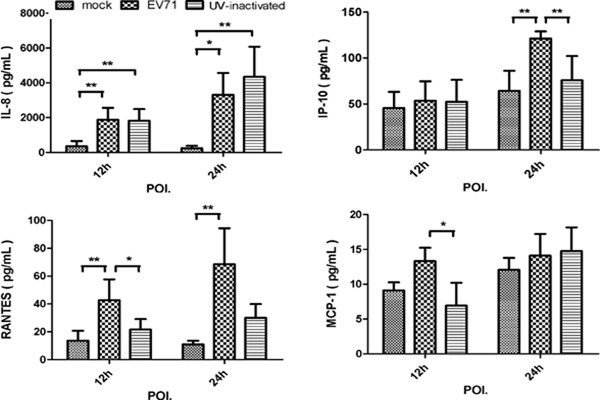
**Chemokines induced in MDMs infected with EV71 and (or) UV-inactivated EV71.** MDMs were infected with or without EV71 (live or UV-inactivated) at MOI of 5 for 12-h and 24-h. The chemokines including IP-10, MCP-1, RANTES, IL-8, MIG concentration in the supernatants at 12-h and 24-h was measured by CBA. Data are expressed as mean ± SEM from eight independent experiments performed in triplicates. (*, P < 0.05 and **, P < 0.01).

Chemokines include a large superfamily and play multiple roles in shaping the innate and adaptive immune responses during viral infection. We measured the levels of the chemokines including CXCL-10/IP-10, CCL-2/MCP-1, CXCL-9/MIG, CXCL-8/IL-8, and CCL-5/RANTES in the supernatants at 12-h and 24-h POI. At early time points (12-h POI.), enhanced production of IL-8 and RANTES was detected in EV71-infected MDMs than in mock-infected MDMs (*p = 0.003*, *p = 0.002*, n = 8), and the concentrations of these chemokines increased along with time (Figure 4). At 24-h POI, much higher concentrations of IL-8, RANTES and IP-10 were found in EV71-infected MDMs than in those of mock( *p = 0.013*, *p = 0.004*, *p = 0.001*, n = 8). Moreover, compared with mock-infected MDMs, UV-irradiated EV71 can induce significantly high level of IL-8 production at 12-h and 24-h POI. than mock (*p = 0.004*, *p = 0.002*, n = 8).

### Up-regulation of TLR2, TLR7 and TLR8 mRNA expression in EV71 infected- MDMs

**Figure 5:**
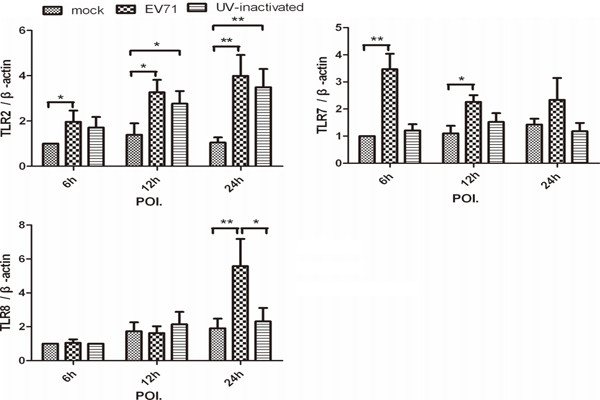
**A higher mRNA expression of TLR2, TLR7 and TLR8 were induced in MDMs infection with EV71.** The mRNA expression of TLR2, TLR3, TLR4, TLR6, TLR7, TLR8, TLR9, TLR10, RIG-1 and MDA-5 were performed by SYBR green real-time RT-PCR. The relative change of mRNA expression was analyzed using △△CT method. To standardize results for variability in cDNA quantity, we expressed them with target gene/β-actin as 1 in mock at 6-h POI. Data are expressed as mean ± SEM from eight independent experiments performed in triplicates (*, *P < 0.05* and **, *P < 0.01*).

In order to investigate the involvement of TLRs and RLHs in EV71-infected MDMs, we screened the TLR2, TLR3, TLR4, TLR6, TLR7, TLR8, TLR9, TLR10, RIG-1, Mda-5 mRNA expression by real-time quantitative RT-PCR. The results showed that the mRNA expression of TLR2 appeared to be significantly enhanced in EV71-infected human MDMs from 6-h to 24-h POI. Increased mRNA expressions of TLR2 were also observed in UV-inactivated virus-infected MDMs at 12-h to 24-h POI. as compared with mock (*p = 0.014*, *p = 0.008,* n = 8). Both TLR7 and TLR8 mRNA expressions were significantly enhanced in EV71-infected MDMs at different time points. In contrast to the findings that higher TLR7 mRNA level was induced at earlier time points (6-h and 12-h POI.), enhanced mRNA expression of TLR8 was observed at a relatively later stage, 24-h POI. (Figure 5). In addition, there was minor change in the mRNA expression of TLR3, TLR4, TLR6, TLR9, TLR10, RIG-1 and Mda-5 in the tested samples.

## Discussion

Macrophages, which are shown to support the infection of various viruses including HIV-1, influenza viruses, and poliovirus and so on, play a critical role in presentation of antigens, pathogen clearance, and induction of inflammation during the early phase of viral infection [21-23]. In this study, both viral gene and antigen of EV71 were detected. The increases of virus yields and the number of viral gene copies were observed in EV71-infected MDMs between the 6-h and 48-h POI. Excess cytokine and chemokine responses of MDMs were triggered by EV71. These findings suggested that macrophages may be not only the target cells but also the effectors during EV71 infection.

It is controversial whether TNF-α was involved in fatal EV71 infection. Significant or minor change of blood TNF-α in EV71 patient with both encephalitis and PE was reported by different clinical studies [24,25]. Significant stronger release of TNF-α from EV71-infected MDMs at 12-h POI than 24-h POI in the study, implying that TNF-α was induced by EV71 infection at early stage and maybe involved in its pathology. Consistent with clinical features of EV71 patients with encephalitis and PE, who are presented with higher levels of the proinflammatory cytokines in blood [9,24], IL-6, IL-1β and TNF-α were induced in EV71-infected MDMs. These proinflammatory cytokines are thought to be the potent pyrogens inducing fever, and the magnitude of febrile response correlates with the level of virus shedding in human and animals [26]. Notably, a transient increase of blood brain barrier (BBB) permeability and its injury were found at early stage of EV71 infection [27]. The pathology may be due to an augmentation of systemic and local TNF-α production, which exhibits detrimental effects by enhancing cell infiltration, cytopathic damage, or functioning as a paracellular pathway for the virus across the BBB [28]. Furthermore, the subsequent responses of acute phase proteins and chemokine activations mediated by IL-6, IL-1β and TNF-α could exacerbate virus induced inflammation and pathology [29]. Therefore, the rapid and strong proinflammatory response of MDMs to EV71 may partially explain the clinical severity.

Macrophages or plasmacytoid DCs, specialized in secreting large amounts of type I IFN after virus infection, play an important role in viral infection. However, minor change of IFN-α in EV71-infected MDMs was detected in our study. It is likely that the 3C protein of EV71 virus inhibits type I interferon activation by viral nuclear acid or RIG-I signalling [30]. Although elevated level of IFN-γ in both plasma and cerebrospinal fluid was found in patients with PE [10], the IFN-γ from EV71-infected MDMs was undetectable here. The discrepancy may be a result of the main cellular source of IFN-γ *in vivo* from activated T and NK cells other than macrophages. A series of IFN-γ-responsive and inflammatory chemokines such as RANTES, IP-10 and IL-8 in MDMs were triggered by EV71 virus. Not only live- but also UV-inactivated EV71 can induce IL-8 releasing from MDMs, which suggested that viral proteins may also be involved in inducing of IL-8. IL-8 is a potent chemoattractant and activator of neutrophils, one of the major immune cells responsible for inflammation of CNS during meningitis or encephalitis [31]. Our *in vitro* findings support the clinical findings that a higher total WBC count, absolute neutrophil count and elevated IL-8 and IP-10 level in patients with BE or PE [11,25].

TLRs and RLHs recognize distinct ligands and trigger host immune response in different virus infection [32]. The recognition of human rhinovirus, human parechovirus 1, rotavirus or coxsackie B virus by different host cells are mediated through elevated TLR2, 7, 8 and (or) Mda5 expression, which induce secretion of proinflammatory cytokines, chemokines, and interferons [16-19]. When TLR2, TLR7 and TLR8 were silenced, there was a considerable decrease in cytokine secretion in human airway epithelial cells with HRV-6 infection [18]. In our study, elevated TLR2, TLR7 and TLR8 expressions as well as increased proinflammatory cytokines and chemokines were observed in EV71-infected MDMs. Furthermore, enhanced IL-8 and TLR2 mRNA expression were also found in UV-inactivated virus treated-MDMs. It is likely that the interaction between TLR2 on cell surface and viral proteins rather than viral RNAs is necessary for the activation of MDMs. Significant up-regulations of mRNA for TLR7 and TLR8 were observed at different time points, and it suggested that there were differential kinetics between TLR7 and TLR8 involvements in EV71-infected MDMs. These results indicated the cytokine productions in EV71-infected MDMs may be partly through the activation of TLR2, TLR7 and (or) TLR8. Further investigations such as gene knockout experiments are needed to determine the exact roles of them.

As documented previously, younger children less than 5-year-old were the most susceptible groups to EV71 and usually presented with severe infection [3]. The underlying mechanisms for the severity remain unknown. And enhanced proinflammatory cytokines and chemokines were indeed found in children patients with encephalitis or PE [9,11,24]. Although there is differential expression level of chemokine receptors on adult and neonatal MDMs [33], one of possible factors for the age-related severity in avian influenza virus infection, a similar cytokines/chemokines profile was found in influenza virus-infected adult and neonatal MDMs and the levels of most of the cytokines/chemokines were comparable [23]. Moreover, the adults can also be infected with EV71 and the clinical severity in adult patients with acute encephalitis was similar to those of EV71 infection in children [34]. Therefore, the findings on adult MDMs infection model here may partially reflect natural *in vivo* infection.

## Conclusions

In summary, effective viral replication in EV71-infected MDMs and excessive inflammatory cytokine and chemokine responses of MDMs to the virus were demonstrated for the first time in our study. The results indicate that macrophages are an important target for EV71, and they can trigger pro-inflammatory response and chemokine response against viral infection. However, inordinate macrophages response may be detrimental to the infected host due to exacerbate virus inflammation and pathology. TLR2, TLR7 and TLR8 may participate in the induction of cytokines/chemokines in EV71-infected MDMs. These data suggested that MDMs may play an important role in the pathogenesis of EV71 infection *in vivo* though more evidence is needed.

## Methods

### Isolation and culture of monocyte-derived macrophages

The study was approved for human subject protection by the Ethics Committee of National Institute for Viral Disease Control and Prevention, China CDC. Following informed consent was written by participants.

Peripheral blood obtained from 8 healthy blood donors aged from 20 to 40 years old. Peripheral blood mononuclear cells (PBMCs) were isolated by a Ficoll-Paque density gradient (Pharmacia Biotech, Uppsala, Sweden) to remove erythrocytes, platelets, and cell debris. Monocytes were isolated by plastic adherence, harvested, counted and seeded on tissue culture plates in RPMI 1640 (Invitrogen Life Technologies, Great Island, NY, USA) medium supplemented with 10% heat-inactivated autologous plasma at 10^6^cells/ml. The purity of monocytes was determined by flow cytometry with anti-CD14 monoclonal antibody (Mab, PharMingen, San Diego, CA, USA) and was consistently above 90%. The monocytes were reseed with 2-5 × 10^5^ cells per well onto a 24 well culture plate, and were allowed to differentiate into MDMs for 10–14 days *in vitro*.

### Infection of MDMs with EV71

Enterovirus 71 virus was propagated in RD cells (obtained from ATCC) in DMEM containing 2% fetal bovine serum (FBS, Invitrogen, Grand Island, NY, USA) and incubated at 35°C with 5%CO_2_. When 80% of the cells showed the typical enteroviral cytopathic effect (CPE), the infected cells were harvested after being frozen and thawed for three times. Cell debris was removed by centrifugation and filtration using a 0.22 μm membrane filter (Millipore, Billerica, CA). EV71 viruses were inactivated using ultraviolet radiation 3000 mj/cm^2^, 30 mins on ice (UV-inactivated EV71). Differentiated MDMs were infected by EV71 and UV-inactivated EV71 at MOI (multiplicity of infection) of 0.1, 0.5, 1 and 5. This is taken to be 0-h point of infection (POI.) for the experiments described below. Virus titters were performed by measuring the 50% tissue culture infective dose (TCID50) on Vero cells and calculated by using the Reed and Muench formula.

### Immunofluorescence assay of viral VP1 protein in infected cells

MDMs were fixed with methanol: acetone (1:1) for 5 min. The cell monolayer was incubated by anti-EV71 VP1 monoclonal antibody (MAB979, Millipore, Billerica, CA) at room temperature for 60 min, and followed by labelling FITC-conjugated goat anti-mouse IgG for 1-h. After being completely washed, the cells were observed under a fluorescence microscope.

### Quantification of mRNA by real-time RT-PCR

Infected MDMs cultured in macrophage serum-free medium (Invitrogen, Grand Island, NY, USA) were harvested at 2-h, 6-h, 12-h, 24-h and 48-h POI. Total RNAs were extracted using QIAGEN RNeasy mini kit(QIAGEN, Hilden, Germany). Reverse transcription was performed on DNase-treated total RNA. The cDNA was synthesized from mRNA with oligo(dT)_12–18_ primer and Superscript II reverse transcriptase (Invitrogen, Grand Island, NY, USA). Specific primers and probes used in the real-time PCR assay were listed in Table 1 and Q-PCR were performed by Rote-Gene 3000 Sequence Detection System (QIAGEN, Hilden, Germany). Viral gene copies were quantified on the basis of a TaqMan Probes fluorescence signal after PCR. We expressed viral gene variability as the number of target gene copies per 10^4^copies of β-actin. The relative changes of other human genes were analyzed by SYBR green real-time PCR. Dissociation curve analysis was performed after each assay, to ensure specific target detection.

**Table 1:** Primer sequences and probes used in real-time PCR assay

**Gene**	**Primer and probe sequence (5′to3′)**	**Product length(bp)**	**GenBank**
EV71	(R) CCCGCTCTGCTGAAGAAACT	89	AF302996
	(F)AGTGATGAGAGTATGATTGAGACACG		
	(P) TCGCACAGCACAGCTGAGACCACTC		
β-actin	(R) CAAGTACTCCGTGTGGATCG	90	NM_001101.3
	(F) GGATGCAGAAGGAGATCACTG		
	(P) CCCTGGCACCCAGCACAATGA		
RIG-1	(R) CCTCTGCACTGTTGCTCAGGAC	192	NM_004585.3
	(F) CTCTTGGCTTCGAGATGGCTTC		
MDA5	(R) ATTGGTGACGAGACCATAACGGATA	196	NM_022168.2
	(F) AGGAGTCAAAGCCCACCATCTG		
TLR2	(R) CACAAAGTATGTGGCATTGTCCAG	158	NM_003264.3
	(F) GTGTTGCAAGCAGGATCCAAAG		
TLR3	(R) AGTGCCGTCTATTTGCCACACA	181	NM_003265.2
	(F) AACAGTGCACTTGGTGGTGGAG		
TLR4	(R) ATGCGGACACACACACTTTCAAATA	143	NM_138554.3
	(F) TTGAGCAGGTCTAGGGTGATTGAAC		
TLR6	(R) AGCCTTCAGCTTGTGGTACTTGTTG	138	NM_006068.2
	(F) CAGAGTGAGTGGTGCCATTACGA		
TLR7	(R) TCTTCAACCAGACCTCTACATTCCA	172	NM_016562.3
	(F) GGAACATCCAGAGTGACATCACAG		
TLR8	(R) GATTGCTGCACTCTGCAATAACTGA	196	NM_138636.3
	(F) GCTGCTGCAAGTTACGGAATGA		
TLR9	(R) CAGGGCCTTCAGCTGGTTTC	97	NM_017442.2
	(F) CCGTGACAATTACCTGGCCTTC		
TLR10	(R) TGTGTTGCAAGATAATTCGTGGAGA	103	NM_001017388.1
	(F) CATGATGGTTGGATGGTCAGATTC		
β-actin	(R) CTAAGTCATAGTCCGCCTAGAAGCA	186	NM_001101.3
	(F) TGGCACCCAGCACAATGAA		

### Measurement of cytokines

Concentration of cytokines from culture supernatants were determined by Cytometric Bead Array(CBA). IL-1β, IL-6, TNF-α, IFN-α, IFN-γ, IP-10, IL-8, RANTES, MCP-1, MIG Flex Set reagents (BD Biosciences, San Diego, CA) were used to measure cytokines by flow cytometry according to the manufacturer’s protocol. The results are presented as the means of assays performed in duplicate wells. Data were analyzed by using FCAP Array 0.1 and BD Cytometric Bead Array 1.4 software assay. The theoretical limits of detection were listed as follows: IL-1β(2.3 pg/ml), IL-6 (1.6 pg/ml), TNF-α (1.2 pg/ml), IFN-α (1.5 pg/ml), IFN-γ(1.8 pg/ml), IP-10 (0.5 pg/ml), IL-8 (1.2 pg/ml), RANTES (0.002 pg/ml), MCP-1 (1.3 pg/ml), MIG (1.1 pg/ml).

### Statistical analysis

Statistical significance was determined by Two-Way ANOVA or the Mann–Whitney rank sum tests. All analyses were performed using the Statistical Package for Social Sciences (SPSS13.0) software (SPSS Inc, IL, USA). A probability *P < 0.05* was considered statistically significant.

## Abbreviations

(EV71): Enter virus; (TLRs): 71Toll-like receptors; (RIG-I): Retinoic acid-inducible gene I; (RLHs): RIG-I-like helicases; (MDMs): Monocyte-derived macrophages; (MDA5): Melamoma differentiation associated gene 5; (HFMD): Hand-foot-and-mouth disease; (BE): Brain stem encephalitis; (PE): Pulmonary edema; (CNS): Central nervous system; (BBB): Blood brain barrier; (MOI): Multiplicity of infection; (CSF): Cerebrospinal fluid; (PBMCs): Peripheral blood mononuclear cells.

## Competing interests

The authors declare that they have no competing interests.

## Authors' contributions

Conceived and designed the experiments: ZD, YJ, XG and JZ. Performed the experiments: XG and JZ. Analyzed the data: XG and JZ. Contributed reagents/materials/analysis tools: WZ, NL, JL and LL. Wrote the paper: XG, JZ, WZ and ZD. All authors read and approved the final manuscript.

## Pre-publication history

The pre-publication history for this paper can be accessed here:

http://www.biomedcentral.com/1471-2334/12/224/prepub
